# Presumptive treatment of fever cases as malaria: help or hindrance for malaria control?

**DOI:** 10.1186/1475-2875-7-132

**Published:** 2008-07-16

**Authors:** Roly D Gosling, Christopher J Drakeley, Alex Mwita, Daniel Chandramohan

**Affiliations:** 1 Department of Infectious and Tropical Diseases, London School of Hygiene and Tropical Medicine, Keppel Street, London, WC1E 7HT, UK; 2 National Malaria Control Program, Ministry of Health, P.O.Box 9083, Dar es Salaam, Tanzania

## Abstract

**Background:**

Malaria incidence has been reported to be falling in several countries in sub-Saharan Africa in recent years. This fall appears to have started before the widespread introduction of insecticide-treated nets. In the new era of calls to eliminate and eradicate malaria in sub-Saharan Africa, exploring possible causes for this fall seem pertinent.

**Presentation of the hypothesis:**

The authors explore an argument that presumptive treatment of fever cases as malaria may have played a role in reducing transmission of malaria by the prophylactic effect of antimalarials and their widespread use. This strategy, which is already in practise is termed Opportunistic Presumptive Treatment (OPT).

**Testing the hypothesis:**

Further comparison of epidemiological indicators between areas with OPT and more targeted treatment is required. If data suggest a benefit of OPT, combining long acting antimalarials that have an anti-gametocyticidal activity component plus using high levels of vector control measures may reduce transmission, prevent resistant strains spreading and be easily implemented.

**Implications of the hypothesis:**

OPT is practised widely by presumptive treatment of fever in health facilities and home management of fever. Improving diagnosis using rapid diagnostic tests and thus reducing the number of doses of antimalarials given may have counter intuitive effects on transmission in the context of elimination of malaria in high to moderate transmission settings.

## Background

There is an increasing body of evidence suggesting that the burden of malaria in sub-Saharan Africa has been decreasing over the past decade and continues to do so (see table 1) [1-7]. The causes of this decline are unclear yet with the current calls for elimination and eradication of malaria[8] the identification of factors causing this decrease, in particular those related to control strategies, would seem paramount.

**Table 1:** Summary of studies describing reduction in malaria in sub-Saharan Africa

**Country** **/region**^reference^	**Years of ** **study**	**Measure of** **reduction**	**Reported** **reduction**	**Reported** **Reasons **
Sub-Saharan Africa^1^	1985 to 1999; 2000 to 2007	Parasite prevalence abstracted from over 2,000 sources	15% (average) reduction	No reason given
Coastal Kenya^2^	1999 to 2007	Malaria specific hospital admissions	63%, 53% and 28% reduction in 3 district hospitals	Malaria control interventions
Ifakara, Tanzania^3^	1995 to 2000	Incidence of malaria in < 5 year old children	Reduced from 0.8 to 0.43 episodes per child per year	Economic improvements, liberalisation of health sector and malaria control interventions
Zanzibar, Tanzania^4^	2003 to 2006	Parasite prevalence	97% reduction	Artemisinin Combination Therapy and Insecticide Treated Nets
Mozambique, South Africa and Swaziland^5^	2000–2004	Parasite prevalence	> 60% fall in parasite prevalence in all 3 zones studied	Indoor residual spraying
Guinea-Bissau^6^	1994 versus 2003/2004	Parasite prevalence	Reduced from 44–79% to 3%	Untreated bed nets and urbanisation
Eritrea^7^	2000 to 2004	Incidence of clinical malaria and case fatality rate reported by health facilities	Decrease in malaria incidence of 83.3% and case fatality by from 0.21 to 0.14%	Climate change and malaria control methods (ITNs, IRS and early case detection and treatment)

To date the decrease in malaria transmission has been documented in a number of countries including Kenya[2], Tanzania[3,4], Mozambique[5], Swaziland[5], South Africa[5], Guinea Bissau[6] and Eritrea[7]. The decrease in cases in Southern Africa coincides with indoor residual spraying (IRS) programmes[5] but for the other reports there is no single clear intervention that could explain the declines. In Kenya it was noted that the decline started before the widespread use of insecticide-treated nets (ITNs)[2]. The decline appears to be broadly continent wide: there has been a reported decrease in the mean prevalence of *Plasmodium falciparum *parasites in cross-sectional surveys from 35% between 1985 and1999 to 20% since 2000[1]. Thus, the decline in malaria predates scaling-up of vector control measures including widespread use of ITNs. During the 1980s and 90s the mainstay of malaria control in Africa was presumptive treatment of fever with an anti-malarial. This strategy has been promoted by the Integrated Management of Childhood Illness (IMCI) and is adopted as policy in many developing countries[9]. In places where transmission is low or decreasing this strategy leads to over-diagnosis of malaria cases[10].

## Presentation of hypothesis

The commonest drugs used for presumptive treatment of fever cases as malaria in the past 50 years of malaria control are chloroquine (CQ) and sulphadoxine-pyrimethamine (SP). Both these drugs have a long half-life and their efficacy and effectiveness for prophylaxis have been clearly documented[11]. Thus, treating most febrile illness with antimalarials, practiced widely in sub-Saharan Africa has lead to a mass and frequently applied treatment against malaria that not only cleared parasitaemia in true cases of malaria but also provided chemoprophylaxis for three weeks in the case of CQ and four weeks in the case of SP. We term this Opportunistic Presumptive Treatment (OPT).

The mechanism of OPT is to effectively treat those who are infected and prevent re-infection in both those who are and in those who are not infected. As most infections occur focally[12,13], correctly treating these individuals clears active infection and prevents new infection in those most at risk of malaria. This in turn reduces the parasite burden in individuals and the infectious reservoir in the community. In low incidence areas, depending on frequency of treatment of fever as malaria, the community may be receiving enough anti-malarial prophylaxis to reduce transmission due to decreased carriage of parasites and thus, cause a gradual decline in malaria burden.

## Testing the hypothesis

The likelihood is that the fall in malaria is multi-factorial with general economic development, climate change and malaria control strategies playing roles and identifying the role OPT has played is complex. However, as treatment of all fever cases remains standard practice in many endemic countries a longitudinal comparison of epidemiological parameters between current practice (OPT) and more targeted treatment of parasite positive febrile cases in the context of other control methods (e.g.ITN) should be undertaken. Strategies for evaluation could be (1) OPT with an ACT or non-ACT for all fever cases without any other obvious cause (current practice); (2) OPT plus an antibiotic for all fever cases; (3) ACT for confirmed cases of malaria (slide or rapid test positive) and OPT with non ACT plus an antibiotic for fever cases confirmed negative for malaria.

OPT has similarities to mass administration of antimalarial drugs (MDA). MDA for limited time periods has been tried for reducing transmission in the past without marked or sustained success[14]. However, OPT, given to individuals at high risk of carrying parasites for several years may have a different outcome than MDA. Since OPT is given to individuals who have a febrile illness an artemesinin in combination with a long-acting partner drug such as amodiaquine, mefloquine or piperiquine could be used. Artemesinins would act to reduce gametocyte carriage[15] while the long-acting partner drug would prevent new blood stage infections and thus new gametocyte production. Alternatively, non-ACTs using a combination of long acting drugs maybe preferred, thus saving the artemesinin component for treatment of proven disease, and these could be combined with gametocidal drugs such as primaquine or tafenoquine.

Programmes using long-acting drugs are at risk of inducing drug resistance. Both CQ and SP are no longer recommended for treating clinical malaria in Africa due to high levels of resistance. Combining long acting drugs and using high levels of vector control measures to reduce transmission of resistant strains may reduce the risk of drug resistance. Studies to evaluate the impact of OPT in combination with vector control measures on transmission of malaria, the emergence of drug resistance and cost-effectiveness in Africa will be complex, large and expensive, and will require several years of follow up time. However, implementation of OPT would be relatively easy as the delivery mechanism is largely in place and the practise of presumptive treatment of fever is common amongst health professionals and the community.

## Implications of the hypothesis

Currently OPT is delivered through the routine health care delivery system in most parts of Africa. Home management of malaria[16] is an extension of OPT in the communities. Due to pressures of over-diagnosis of malaria which has adverse outcomes for patients[10] and misinforms malaria control programmes, introduction of rapid diagnostic tests are being considered as a cost effective measure to improve the accuracy of diagnosis of malaria[17]. If the hypothesis is true, restricting antimalarial treatment to confirmed cases of malaria only will have little impact on control in high/moderate endemic settings as fewer people will be exposed to prophylactic levels of drugs. Treating fever cases confirmed as malaria with ACT and as non malaria with non-ACT OPT plus an antibiotic would circumvent this problem and have the added advantage of broadening syndromic management of fever to cover the most serious alternative diagnoses (i.e. bacterial sepsis). However, any widespread use of drugs will result in the community being exposed to drugs and their associated problems of side effects and drug resistance.

As transmission decreases there will be a point at which there is no benefit to be gained from treating asymptomatic carriers and preventing infection by prophylaxis. The level of transmission where changing strategies becomes advantageous is not known. However, when this point is reached active case detection and treatment plus surveillance for outbreaks would be the control method of choice.

Malaria remains a huge public health issue that needs the full armoury of current interventions. The good news is that, for whatever reason, malaria seems to be decreasing in many parts of sub-Saharan Africa. The recent call for eradication of malaria is timely and to achieve this aim all effective interventions need to be scaled up. In areas of moderate to high transmission mass over-treatment of fever as malaria (OPT) should be investigated as tool for malaria control. The major risk of this strategy is increasing drug resistance but the benefits may be much reduced malaria as is evident in parts of Africa.

## Authors' contributions

All authors discussed and reviewed the ideas presented in the paper. All authors wrote the paper and read and approved the final version.
